# Hayman’s diallel analysis for yield-related traits in F_1_ and F_2_ durum wheat (*Triticum durum* Desf.) progenies

**DOI:** 10.1371/journal.pone.0342977

**Published:** 2026-02-24

**Authors:** Insaf Bentouati, Abderrahmane Hannachi, Zine El Abidine Fellahi, Abdelhamid Mekhlouf, Aleksandra O. Utkina, Mohamed S. Shokr, Nazih Y. Rebouh

**Affiliations:** 1 Department of Agronomic Sciences, Faculty of Natural and Life Sciences, University of Ferhat Abbas Sétif 1, Algeria; 2 Laboratory for the Improvement and Development of Plant and Animal Production, University of Ferhat Abbas Sétif-1, Sétif, Algeria; 3 National Agronomic Research Institute of Algeria (INRAA), Sétif Research Unit, Algeria; 4 Department of Agronomic Sciences, Faculty of Natural, Life and Earth Sciences and the Universe, University of Mohamed El Bachir El Ibrahimi, Bordj Bou Arréridj, Algeria; 5 Laboratory for the Valorization of Natural Biological Resources, University of Ferhat Abbas Sétif-1, Sétif, Algeria; 6 Institute of Environmental Engineering, RUDN University, Moscow, Russia; 7 Soil and Water Department, Faculty of Agriculture, Tanta University, Tanta, Egypt; Nuclear Science and Technology Research Institute, IRAN, ISLAMIC REPUBLIC OF

## Abstract

This study employed Hayman’s diallel analysis to investigate the inheritance patterns of eight agronomic traits in durum wheat (*Triticum durum* Desf.) using a 4 × 4 half-diallel mating design evaluated across F_1_ and F_2_ generations. Field trials were conducted at the INRAA experimental station in Sétif, Algeria, during the 2021–2022 and 2023–2024 growing seasons. The analysis revealed significant genotypic variation across all studied traits, with distinct inheritance patterns emerging between generations. Plant height was predominantly governed by additive gene action. In contrast, spike length and number of grains per spike shifted from overdominance in F_1_ to partial dominance in F_2_, reflecting enhanced additive effects post-recombination. Yield components, including spike weight, number of spikes per plant, and grain yield, exhibited persistent non-additive inheritance and overdominance across generations, indicating limited early-generation selection efficiency. Dominance effects were significant in F_1_ but diminished in F_2_ for most traits, suggesting a recombination-mediated breakdown of heterotic patterns. Allele distribution was asymmetric, highlighting unequal parental contributions and the potential for heterosis exploitation. Most traits were controlled by a single gene or a closely linked gene block, as indicated by the ${\hat{h}}^{\text{2}}/{\hat{H}}_{\text{2}}$ ratio. High broad-sense heritability, in contrast to variable narrow-sense heritability, suggests the need for generation-specific breeding strategies. These findings support pedigree selection for additive traits in early generations and recurrent or advanced-generation selection for yield components, thereby optimizing durum wheat improvement under semi-arid Mediterranean conditions.

## 1. Introduction

Durum wheat (*Triticum durum* Desf.) is a globally significant cereal crop, esteemed for its high protein content and superior semolina quality, making it indispensable in the production of pasta and other wheat-based products. In countries like Algeria, durum wheat is not only a staple food source but also a cornerstone of food security, economic stability, and rural livelihoods, particularly in semi-arid regions where it is predominantly cultivated [1].

The escalating global population and shifting consumer preferences have intensified the demand for wheat production. Consequently, wheat breeders are under increasing pressure to enhance crop yields, improve grain quality, and develop varieties that are resilient to pests, diseases, and environmental stresses [2,3]. Genetic analysis techniques, such as diallel analysis, have become pivotal in plant breeding, offering profound insights into the inheritance patterns of traits, elucidating genetic interactions, and facilitating the development of superior crop varieties [4,5].

Diallel mating designs remain instrumental in plant breeding, enabling the development of superior varieties with enhanced characteristics across both self-pollinated and cross-pollinated crops. These designs would allow breeders to identify optimal parental combinations that produce offspring with desirable traits and to comprehend the genetic architecture underlying complex traits [6].

Among the various analytical models available for diallel cross data, Griffing’s and Hayman’s methods are prominently utilized by [7–9]. Griffing’s methodology offers a robust framework for dissecting the genetic basis of quantitative traits, predicting hybrid performance, and selecting optimal parents for breeding programs. This approach is widely used in wheat improvement to develop varieties with enhanced characteristics, including higher yield potential, disease resistance, and improved quality parameters. Griffing’s method enables breeders to estimate genetic parameters, particularly the components of genetic variance, involved in trait inheritance, and to determine the contributions of general combining ability (GCA) and specific combining ability (SCA) in trait expression [5,10].

Hayman’s method is another valuable statistical approach that provides a relatively straightforward and efficient means to estimate genetic parameters in diallel cross analysis. It offers comprehensive genetic information, including additive and dominance effects of genes, the nature and direction of dominance, gene distribution among parents, maternal and reciprocal effects, the ratio of dominant to recessive alleles in parents, and heritability estimates [7,8]. However, the proper application of this method requires certain assumptions: the absence of epistasis, maternal effects, multiple allelism, independent gene distribution between parents, diploid segregation, and homozygous parents [7,8,11,12]. Breeders must carefully consider these assumptions when making breeding decisions, as they inform the selection of parental lines and breeding strategies for developing new crop varieties with desired traits.

Hayman’s method can be implemented both graphically and numerically to understand trait inheritance in hybrid populations resulting from crosses between different parental genotypes. The graphical approach involves plotting the covariance of each array against its variance, allowing for a visual assessment of gene action and genetic interactions. The slope and position of the regression line fitted to the array points within the limiting parabola reveal the nature of gene action and the presence or absence of gene interaction. The average degree of dominance can be inferred from the position of the regression line on the ${\hat{W}}_{r}+{\hat{V}}_{r}$ graph [4,5]. The numerical approach involves fitting a regression model to the data, providing precise estimates of genetic parameters such as additive genetic variance, non-additive genetic variance, and gene interactions, which are crucial for further analysis and decision-making in breeding programs [11]. The choice between graphical and numerical approaches depends on the data collected; however, combining both methods provides a comprehensive understanding of diallel experimental results.

While diallel analyses in durum wheat are well documented, most studies provide only a static snapshot of gene action, predominantly in the F_1_ generation. This creates a notable knowledge gap, as the transition from the hybrid F_1_ to the segregating F_2_ generation is a critical phase in breeding programs [13]. Moreover, the escalating challenges of climate change underscore the need to understand the genetic architecture of complex traits under realistic stress conditions. Gene action is highly context-dependent, and diallel studies on Algerian durum wheat under semi-arid conditions remain limited, underscoring the need to generate locally relevant insights to inform breeding decisions.

To address these gaps, this study uses Hayman’s diallel analysis to quantify and compare the genetic components of yield-related traits in F_1_ and F_2_ durum wheat progenies under Algerian semi-arid conditions and to translate these insights into practical breeding strategies. The results are expected to strengthen knowledge of durum wheat genetics and guide breeders in improving trait expression and productivity in Mediterranean environments.

## 2. Materials and methods

### 2.1. Plant material and experimental design

This study was conducted using four genetically diverse durum wheat (*Triticum durum* Desf.) cultivars selected for their contrasting phenotypes in plant height and yield-related traits (Table 1). These cultivars were sourced from international breeding programs, including the International Center for Agricultural Research in the Dry Areas (ICARDA) and the International Maize and Wheat Improvement Center (CIMMYT), as well as local Algerian germplasm.

**Table 1:** Parental wheat cultivars, their pedigrees, and origins used in the study.

Genotype	Code	Pedigree	Origin
Achouri	P1	*Mrf1/Stj2//Gdr2/Mgnl1*	ICARDA
Waha	P2	*Plc/Ruff//Gta’s/3/Rolette CM 17904*	CIMMYT-ICARDA
Beni Mestina	P3	*Lahn/cham12003*	ICARDA
Mohamed Ben Bachir	P4	*Landrace*	Algeria

The four parental genotypes were crossed in a 4 × 4 half-diallel mating scheme during the 2020–2021 cropping season, resulting in six F_1_ hybrids. The F_1_ progenies and parental lines were evaluated during the 2021–2022 season, while the corresponding F_2_ populations were assessed in the 2023–2024 season. The field experiments were conducted at the experimental station of the National Agronomic Research Institute of Algeria (INRAA), located in Sétif, a central region for cereal production. Trials were laid out in a randomized complete block design (RCBD) with three replications to minimize environmental variability and enhance statistical reliability. Standard agronomic practices, including timely sowing, fertilizer application, and weed management, were uniformly applied in accordance with national wheat production guidelines.

Data were collected on a single-plant basis for eight morpho-agronomic traits, including plant height (PH), spike length (SL), spike weight (SW), number of spikes per plant (NS), number of grains per spike (NGS), thousand kernel weight (TKW), grain yield (GY), and above-ground biomass (BIO). These traits were recorded for both F_1_ ((S1 Table in S1 File) and F_2_ (S2 Table in S1 File) generations, allowing for the estimation of genetic parameters across successive generations.

### 2.2. Statistical analyses

Data were initially subjected to analysis of variance (ANOVA) following the procedures described by Singh and Chaudhary [4], to assess the significance of differences among genotypes. For traits exhibiting significant genotypic variation, Hayman’s diallel [7,8] was implemented to dissect the genetic architecture. This biometrical method relies on several assumptions: diploid chromosome segregation, homozygous parents, absence of reciprocal effects, no epistasis, absence of multiple allelism, and independent gene distribution among parents. Before genetic analysis, these assumptions were verified through a series of diagnostic tests.

#### 2.2.1. Model adequacy testing.

The validity of the additive-dominance model was evaluated using Hayman’s approach [7,8], supported by four complementary tests as outlined by Singh and Chaudhary [4] and Cruz et al. [5].

1. Regression coefficient test: A regression of parent-offspring covariance (${\hat{W}}_{r}$) on array variance (${\hat{V}}_{r}$) was performed, and the slope (*b*) was tested against the null hypothesis *H*_*0*_*: b=1* using a *t*-test (α = 0.05). Significant deviation from unity indicated model inadequacy.
2. Rotated axes test: Following a 45° rotation of the ${\hat{W}}_{r}-{\;\hat{V}}_{r}$ coordinate system, the new slope (*b′*) was tested against *H*_*0*_*: b′=0* using an *F*-test (α = 0.05). A significant *F*-value suggested a violation of model assumptions.
3. ANOVA of arrays (${\hat{W}}_{r}+{\;\hat{V}}_{r}$): Significant between-array variation for (${\hat{W}}_{r}+{\;\hat{V}}_{r}$) indicated the absence of epistatic allelic interactions, supporting model adequacy.
4. ANOVA of arrays (${\hat{W}}_{r}-{\;\hat{V}}_{r}$): Non-significant between-array variation for (${\hat{W}}_{r}-{\;\hat{V}}_{r}$) confirmed the presence of dominance without confounding non-allelic interactions.

Traits satisfying all four tests were considered fully adequate for the additive-dominance model. Those failing only one test were classified as partially adequate and retained for analysis with appropriate caution in interpretation. Traits failing multiple tests were excluded from genetic parameter estimation.

#### 2.2.2. Genetic component estimation.

For traits that met full or partial model adequacy, generation-specific genetic components were estimated using diallel analysis based on Hayman’s approach [7,8] and Mather’s concepts of additive ($\hat{D}$) and dominance (${\hat{H}}_{\text{1}}$ and ${\hat{H}}_{\text{2}}$) variance components [12]. The F_1_ genetic components were calculated according to Hayman’s original formulas, while the F_2_ variance components were estimated using modified formulas proposed by [4,14,15]. Additive genetic variance ($\hat{D}$): $\hat{D}=V_{\text{0}L\text{0}}–\hat{E}$, where *V*_*0L0*_ represents the variance of the parental lines, and $\hat{E}$ denotes the expected environmental component of variation.

1. Dominance genetic variance (${\hat{H}}_{\text{1}}$): ${\hat{H}}_{\text{1}}=\;V_{\text{0}L\text{0}}\;–\;\text{4}W_{\text{0}L\text{0}\text{1}}\;+\;\text{4}V_{\text{1}L\text{1}}–((\text{3}n–\text{2})\hat{E})/n$) for F_1_ and ${\hat{H}}_{\text{1}}\;=\;\text{1}\text{6}V_{\text{1}L\text{2}}\;–\;\text{1}\text{6}W_{\text{0}L\text{0}\text{2}}+\text{4}V_{\text{0}L\text{0}}–(\text{4}(\text{5}n-\text{4})\hat{E})/n$ for F_2_, where *W*_*0L01*_ is the mean covariance between parents and their arrays, *V*_*1L1*_ is the mean variance of the arrays. n is the number of parental genotypes.
2. Dominance genetic variance (${\hat{H}}_{\text{2}}$): ${\hat{H}}_{\text{2}}=\;\text{4}V_{\text{1}L\text{0}}\;–\;\text{4}V_{\text{0}L\text{0}}\;–\;\text{2}\hat{E}$ for F_1_ and ${\hat{H}}_{\text{2}}=\;\text{1}\text{6}V_{\text{1}L\text{2}}\;–\;\text{1}\text{6}V_{\text{0}L\text{2}}\;–\;(\text{1}\text{6}(n–\text{1})\hat{E})/n$) for F_2_, where *u* and *v* represent the proportions of positive and negative alleles in the parents, respectively.
3. Dominance effect (${\hat{h}}^{\text{2}}$): ${\hat{h}}^{\text{2}}=\;\text{4}(M_{L\text{1}}\;–M_{L\text{0}})²\;–(\text{4}(n\;–\text{1})\hat{E})/n²$ for F_1_ and ${\hat{h}}^{\text{2}}\;=\;(\text{4}M_{L\text{2}}–\text{4}M_{L\text{0}})²–(\text{1}\text{6}(n–\text{1})\hat{E})/n$ for F_2_, where, *M*_*L1*_ and *M*_*L0*_ represent the total and diagonal values, respectively. The significance of ${\hat{h}}^{\text{2}}$ supports unidirectional dominance, whereas the non-significant value of ${\hat{h}}^{\text{2}}$ suggests that a substantial contribution to dominance was due to the homogeneity of loci. When dominant and recessive alleles are equally frequent, ${\hat{H}}_{\text{1}}$= ${\hat{H}}_{\text{2}}$ = ${\hat{h}}^{\text{2}}$.
4. Covariance of additive and dominance effects ($\hat{F}$): $\hat{F}=\;\text{2}V_{\text{0}L\text{0}}\;–\;\text{4}W_{\text{0}L\text{0}\text{1}}\;–\;(\text{2}(n\;–\text{2})\hat{E})/n$ for F_1_ and $\hat{F}\;=\;\text{4}V_{\text{0}L\text{0}}\;–\text{8}W_{\text{0}L\text{0}\text{2}}–(\text{4}(n–\text{2})\hat{E})/n$ for F_2_. A positive $\hat{F}$ indicates dominant alleles are more prevalent among parents, while a negative value implies recessive allele prevalence. $\hat{F}\;=\;\text{0}$ suggests either the absence of dominance or equal distribution of dominant and recessive alleles.
5. Environmental variance component ($\hat{E}$): $\hat{E}$ is the expected environmental component of variation

Significance of genetic components in F_1_ was assessed using the standard error test (value/SE > 1.96), while F_2_ components were evaluated using *t*-tests with (n−2) degrees of freedom.

#### 2.2.3. Derived genetic parameters.

From the primary genetic components, the following secondary parameters were calculated:

1. Average degree of dominance: $\left({\hat{H}}_{\text{1}}/\;\;\;\;\hat{D}\right)$ for F_1_ and $({¼\hat{H}}_{\text{1}}/\;\hat{D}$) for F_2_. Values below 1 indicate partial dominance; equal to 1 suggests complete dominance, and values above 1 imply overdominance.
2. Frequency of alleles with positive and negative effects: ${\hat{H}}_{\text{1}}/{\text{4}\hat{H}}_{\text{1}}$. A value of 0.25 reflects an equal distribution of positive and negative alleles among parents.
3. Proportion of dominant/recessive alleles: $\hat{K}/{\;\hat{K}}_{R}=[(\text{4}\;\hat{D}{\hat{H}}_{\text{1}})+\hat{F}]/[(\text{4}\;\hat{D}{\hat{H}}_{\text{1}})–\hat{F}]$ for F_1_ and ${\hat{K}}_{D}/{\;\hat{K}}_{R}=[¼(\text{4}\;\hat{D}{\hat{H}}_{\text{1}}+\;½\;\hat{F}]/[¼(\text{4}\hat{D}{\hat{H}}_{\text{1}})\;–\;½\;\hat{F}]$ for F_2_. A ratio of 1 indicates a balanced distribution of dominant and recessive alleles. Values less than 1 suggest an excess of recessive alleles, whereas values greater than 1 suggest a predominance of dominant alleles.
4. Number of gene groups exhibiting dominance: ${\hat{h}}^{\text{2}}/{\hat{H}}_{\text{2}}$. This metric provides an approximation of the number of loci or gene groups controlling the trait that express dominance effects.
5. Broad-sense heritability: $h_{bs}^{\text{2}}=[½D+½{\hat{H}}_{\text{1}}–¼{\hat{H}}_{\text{2}}–½\;\hat{F}]/\;[½D+½{\hat{H}}_{\text{1}}\;–¼{\hat{H}}_{\text{2}}–½\;\hat{F}+\hat{E}]$.
6. Narrow-sense heritability: $h_{ns}^{\text{2}}=[½\hat{D}+½{\hat{H}}_{\text{1}}–½{\hat{H}}_{\text{2}}–½\;\hat{F}]/\;[½D+½{\hat{H}}_{\text{1}}\;–¼{\hat{H}}_{\text{2}}–½\;\hat{F}+\hat{E}]$ for F_1_ and $h_{ns}^{\text{2}}=¼\hat{D}/[¼D+\frac{\text{1}}{\text{1}\text{6}}{\hat{H}}_{\text{1}}\backslash -\hat{F}+\hat{E}]$ for F_2_.

#### 2.2.4. Graphical analysis.

The regression of parent-offspring covariance (${\hat{W}}_{r}$) on array variance (${\hat{V}}_{r}$) provided visual insights into dominance relationships and allele distribution patterns (Full dataset are provided in (S3 Table in S1 File) [5]. Completely recessive parents cluster at the upper end of the regression line, while thoroughly dominant parents appear at the lower intersection with the limiting parabola. In F_1_ populations, complete dominance (${\hat{H}}_{\text{1}}=\hat{D}$) produces a regression intercept at the origin; partial dominance shifts the intercept above the origin, and overdominance places it below. In case of no dominance (${\hat{H}}_{\text{1}}=\text{0}$), the regression line is tangent to the parabola. In F_2_ populations, the interpretation is adjusted to account for the inbreeding-related halving of dominance effects. Complete dominance now corresponds to $\text{4}\hat{D}={\hat{H}}_{\text{1}}$, and an intercept at the origin indicates overdominance. For partial dominance, the intercept’s position along the AB segment becomes critical: the midpoint (½AB) marks the threshold for complete dominance, with intercepts above signifying partial dominance and those below indicating overdominance. Detection of overdominance in F_2_ thus requires the stricter condition $\hat{D}<¼{\hat{H}}_{\text{1}}$ [4].

The correlation between (${\hat{W}}_{r}+{\hat{V}}_{r}$) and phenotypic means (${\hat{Y}}_{r}$) was also analyzed to infer the directional distribution of dominant and recessive alleles. A significant negative correlation implies that favorable alleles are predominantly dominant, whereas a positive correlation indicates that favorable alleles are largely recessive. The relative dispersion of array points in the graph provides options for improving hybridization [8].

All statistical analyses were conducted using Genes software [16], with F_2_-specific adjustments implemented in Microsoft Excel Worksheets, as described by Singh and Chaudhary [4].

## 3. Results

### 3.1. Analysis of variance

Analysis of variance (ANOVA) revealed significant differences among genotypes for all evaluated agronomic traits in both F_1_ and F_2_ generations, indicating substantial genetic variability (Table 2).

**Table 2:** Mean squares for agronomic measured traits in durum wheat.

	F_1_/F_2_	Mean squares					
		Blocs	Genotypes	Parents	F_1_/F_2_	Parents vs. F_1_/F_2_	Error
**Df**	**F**_**1**_	2	9	3	6	1	18
	**F**_**2**_	2	9	3	6	1	18
**PH**	**F**_**1**_	3.98	669.14**	1109.90**	455.22**	138.67^ns^	23.61
	**F**_**2**_	58.18	196.78**	363.46**	75.85^ns^	100.46^ns^	50.49
**SL**	**F**_**1**_	0.16	1.62**	1.50**	1.42**	0.98^ns^	0.09
	**F**_**2**_	0.01	1.17**	2.24**	0.72**	0.07^ns^	0.03
**SW**	**F**_**1**_	4.99	130.42**	86.62^ns^	78.42**	173.96*	16.01
	**F**_**2**_	0.63	2.03**	1.04^ns^	3.00**	0.04^ns^	0.31
**NS**	**F**_**1**_	0.34	38.75**	35.76*	18.65**	49.39*	3.29
	**F**_**2**_	0.30	0.79**	0.75*	0.95*	0.03^ns^	0.18
**NGS**	**F**_**1**_	1.45	64.87**	8.11*	50.51**	102.34*	2.18
	**F**_**2**_	4.35	67.67**	104.95**	45.88**	21.51^ns^	5.16
**TKW**	**F**_**1**_	2.76	41.18**	40.79*	28.51^ns^	35.22^ns^	6.79
	**F**_**2**_	1.99	177.05**	308.69**	133.01**	0.75^ns^	20.04
**GY**	**F**_**1**_	2.31	55.35**	73.40**	23.47**	53.54^ns^	2.79
	**F**_**2**_	0.32	3.40**	2.76**	3.19**	2.12^ns^	0.38
**BIO**	**F**_**1**_	21.86	393.77**	174.02^ns^	309.26**	491.83*	27.53
	**F**_**2**_	0.71	6.28**	2.15^ns^	7.67**	3.91^ns^	1.14

PH: Plant height, SL: Spike length, SW: Spike weight, NS: Number of spikes plant^-1^, NGS: Number of grains spike^-1^, TKW: Thousand kernel weight, GY: Grain yield, BIO: Above-ground biomass. Df: degrees of freedom, ns, * and **: non-significant and significant at 0.05 and 0.01 probability levels, respectively.

In both generations, significant to highly significant variation was observed among parental lines for PH, SL, NS, NGS, TKW, and GY. However, SW and BIO did not show significant differences among parents but displayed highly substantial variation among F_1_ and F_2_ progenies. In the case of PH, highly significant differences were noted among F_1_ progenies, whereas the variation became non-significant in F_2_. The parents vs. F_1_ contrast was significant for SW, NS, and BIO but not for PH, SL, or TKW. In contrast, the parents vs. F_2_ contrast was non-significant for all traits, including those (SW, NS, BIO) that showed significant differences between parents and F_1_.

### 3.2. Sufficiency test of the additive-dominant model

The adequacy of the additive-dominance model varied across traits and filial generations (Table 3). In the F_1_ generation, the model was fully adequate for PH, SL, NS, GY, and BIO. However, only partial adequacy was observed for SW, NGS, and TKW. For NGS, a significant deviation of the regression coefficient ($\hat{b}$) from unity and a significant *F*-test for the rotated regression slope (*b′*) were noted. In SW and TKW, the non-significant mean squares for the (${\hat{W}}_{r}+{\hat{V}}_{r}$) arrays indicated that the additive-dominance model did not fully explain the inheritance patterns. In the F_2_ generation, the additive-dominance model was fully adequate for SW, NS, NGS, and TKW. In contrast, PH, SL, GY, and BIO showed only partial adequacy. Specifically, the (${\hat{W}}_{r}+{\hat{V}}_{r}$) arrays were non-significant in PH, SL, and BIO. Significant mean squares for the (${\hat{W}}_{r}{-\hat{V}}_{r}$) arrays were found in GY and BIO. Additionally, significant deviations of the regression coefficient ($\hat{b}$) from unity were observed in PH and GY.

**Table 3:** Sufficiency test of the additive-dominant model based on the linear regression analysis of ${\hat{W}}_{r}$ in ${\;\hat{V}}_{r}$ for agronomic measured traits in durum wheat.

Traits	F_1_/F_2_	ANOVA of arrays		Regression (${\hat{W}}_{r}=\hat{a}+\hat{b}{\hat{V}}_{r}$)			Model adequacy
		${\hat{W}}_{r}$ – ${\hat{V}}_{r}$	${\hat{W}}_{r}$ + ${\hat{V}}_{r}$	$\hat{b}$ ± *Variance*	*t* (*H*_*0*_*: b*_*1*_ = 1)	*F = t²* *(H*_*0*_ *: b’0 = b – 1 = 0)*	
**PH**	**F**_**1**_	15101.85^ns^	131706.83*	0.70 ± 0.13	–0.84 ^ns^	0.35^ns^	Fully adequate
	**F**_**2**_	5187.06^ns^	15466.28^ns^	0.43 ± 0.08	–2.06*	1.19^ns^	Partially adequate
**SL**	**F**_**1**_	0.02^ns^	0.73*	0.86 ± 0.04	–0.72 ^ns^	0.48^ns^	Fully adequate
	**F**_**2**_	0.003^ns^	0.05^ns^	0.96 ± 0.25	–0.08 ^ns^	–0.42 ^ns^	Partially adequate
**SW**	**F**_**1**_	2702.36^ns^	13555.70^ns^	0.43 ± 0.13	–1.61 ^ns^	0.79^ns^	Partially adequate
	**F**_**2**_	0.27^ns^	1.22*	0.45 ± 0.34	–0.95 ^ns^	0.11^ns^	Fully adequate
**NS**	**F**_**1**_	148.32^ns^	1661.30*	0.60 ± 0.10	–1.25 ^ns^	0.68^ns^	Fully adequate
	**F**_**2**_	0.04^ns^	0.38*	1.17 ± 0.02	1.32^ns^	–1.56 ^ns^	Fully adequate
**NGS**	**F**_**1**_	199.60^ns^	469.64*	0.08 ± 0.02	–6.01*	3.08*	Partially adequate
	**F**_**2**_	12.93^ns^	1.23*	0.82 ± 0.06	–0.75 ^ns^	0.45^ns^	Fully adequate
**TKW**	**F**_**1**_	112.45^ns^	636.73^ns^	1.00 ± 0.20	–0.01 ^ns^	–0.47 ^ns^	Partially adequate
	**F**_**2**_	7413.53^ns^	30122.59*	0.83 ± 0.44	–0.26 ^ns^	–0.42 ^ns^	Fully adequate
**GY**	**F**_**1**_	334.15^ns^	3667.86*	0.69 ± 0.10	–0.99 ^ns^	0.52^ns^	Fully adequate
	**F**_**2**_	2.69*	4.70*	–0.70 ± 0.50	–2.42*	–0.35 ^ns^	Partially adequate
**BIO**	**F**_**1**_	23446.01^ns^	117018.0*	0.53 ± 0.10	–1.51 ^ns^	0.85^ns^	Fully adequate
	**F**_**2**_	9.75*	11.26^ns^	0.69 ± 0.38	–0.50 ^ns^	–0.20 ^ns^	Partially adequate

PH: Plant height, SL: Spike length, SW: Spike weight, NS: Number of spikes plant^-1^, NGS: Number of grains spike^-1^, TKW: Thousand kernel weight, GY: Grain yield, BIO: Above-ground biomass. ns and *: non-significant and significant at 0.05 probability level, by *F* test and by *t* test.

### 3.3. Additive and dominance effects across generations

#### 3.3.1. Plant height.

**Fig 1:**
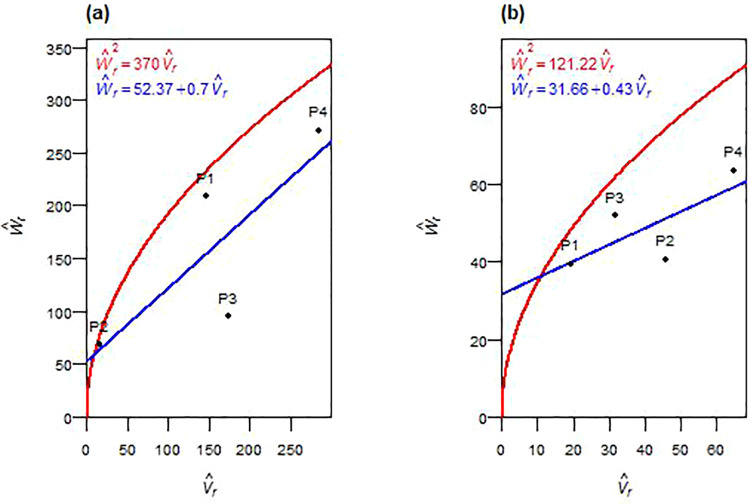
${\hat{W}}_{r}-{\hat{V}}_{r}$ graphs for plant height in F_1_ (a) and F_2_ (b) durum wheat.

The inheritance of plant height showed consistent genetic patterns across both F_1_ and F_2_ generations (Tables 4 and 5). Additive genetic variance ($\hat{D}$) was significant in both generations, while dominance components (${\hat{H}}_{\text{1}}$ and ${\hat{H}}_{\text{2}}$) remained non-significant. Dominance deviation (${\hat{h}}^{\text{2}}$) was significant in F_2_. Partial dominance was observed in both generations, as evidenced by average dominance degrees ($\left({\hat{H}}_{\text{1}}/\hat{D}\right)$ < 1 in F_1_ and $\left(¼{\hat{H}}_{\text{1}}/\hat{D}\right)$ < 1 in F_2_), and the regression of ${\hat{W}}_{r}$ on ${\;\hat{V}}_{r}$ intercepted above the origin (Fig 1a, b). Asymmetric allelic distribution (${\hat{H}}_{\text{2}}/\text{4}{\hat{H}}_{\text{1}}$ ≠ 0.25) was found in both generations. Positive $\hat{F}$ values and ${\hat{K}}_{D}/{\hat{K}}_{R}$ ratios > 1 confirmed a predominance of dominant alleles. The ratio ${\hat{h}}^{\text{2}}/{\hat{H}}_{\text{2}}$ implied the involvement of approximately one dominant gene or gene block. In F_1_, a high positive correlation (*r*(${\hat{W}}_{r}$+ ${\;\hat{V}}_{r}$,  ${\;\hat{Y}}_{r}$)) was observed. Parental genotype P2 had the most dominant alleles, being closest to the origin, while P4 was farthest, containing maximum recessive alleles (Fig 1a). In F_2_, a negative *r(*${\hat{W}}_{r}$+ ${\;\hat{V}}_{r}$,  ${\;\hat{Y}}_{r}$) correlation was recorded, with P4 showing the highest concentration of recessive alleles and P1 had the most dominant alleles (Fig 1b). Environmental variance ($\hat{E}$) increased significantly in F_2_. Heritability estimates (*h²*_*bs*_ and *h²*_*ns*_) were high across both generations.

**Table 4:** Estimates of genetic components for agronomically measured traits in durum wheat.

Traits	F_1_/F_2_	Estimates ± standard errors^#^						
		$\hat{D}$	${\hat{H}}_{1}$	${\hat{H}}_{2}$	${\hat{h}}^{2}$	$\hat{F}$	$\hat{E}$	$\hat{D}$ *–* ${\hat{H}}_{1}$
**PH**	**F**_**1**_	346.39*	255.67	233.89	107.91	74.35	23.61	90.72
	**F**_**2**_	104.13*	75.69	100.42	171.42*	59.19	17.09*	28.44
**SL**	**F**_**1**_	0.41*	0.77*	0.60*	0.82*	–0.15	0.09*	–0.36
	**F**_**2**_	0.73*	0.40	0.32	0.13	0.06	0.01	0.33
**SW**	**F**_**1**_	12.86	100.06	90.23	147.91*	11.62	16.01	–87.20
	**F**_**2**_	0.23	11.17*	7.75*	–1.25	0.97	0.12	–10.94
**NS**	**F**_**1**_	8.64	32.81	29.59	43.26*	7.85	3.29	–24.17
	**F**_**2**_	0.32*	2.17*	1.57	–0.48	–0.21	0.06	–1.86
**NGS**	**F**_**1**_	0.53	74.96*	65.43*	93.95*	–2.80	2.18	–74.44
	**F**_**2**_	33.35*	71.21*	58.13*	60.27*	1.28	1.69	–37.86
**TKW**	**F**_**1**_	6.79*	8.41	10.35	26.62*	–3.80	6.79*	–1.63
	**F**_**2**_	96.84*	1002.14*	802.85*	–70.17	273.43	6.08	–905.30
**GY**	**F**_**1**_	21.69*	47.34	40.41	47.62*	18.99	2.79	–25.64
	**F**_**2**_	0.80	22.89*	18.49*	6.45	3.69	0.13	–22.09
**BIO**	**F**_**1**_	30.48	308.40	295.81	435.42*	–46.91	27.53	–277.92
	**F**_**2**_	0.35	20.32	18.86	10.30	–2.89	0.37	–19.97

PH: Plant height, SL: Spike length, SW: Spike weight, NS: Number of spikes plant^-1^, NGS: Number of grains spike^-1^, TKW: Thousand kernel weight, GY: Grain yield, BIO: Above-ground biomass. $\hat{D}$: Variance compound due to additive effects, ${\hat{H}}_{\text{1}}$: Variance compound due to non-additive genic effects, ${\hat{H}}_{\text{2}}$: genetic variance compound for the non-additive genic effects corrected by the genic distribution, ${\hat{h}}^{\text{2}}$: Quadratic compound determined by the mean difference between hybrids and parents, $\hat{F}$: Covariance genetic compound between additive and non-additive genic effects, $\hat{E}$: Environmental variance compound, $\hat{D}-{\hat{H}}_{\text{1}}$: Compound that expresses the difference between additive and dominant genic effects. *: significant at 0.05 level of probability.

**Table 5:** Ratios of genetic parameters for agronomically measured traits in durum wheat.

Components	PH		SL		SW		NS	
	F_1_	F_2_	F_1_	F_2_	F_1_	F_2_	F_1_	F_2_
**F**_**1**_: $({\hat{H}}_{1}/\hat{D}$***)*** **F**_**2**_: $(¼{\hat{H}}_{1}/\hat{D})$	0.86	0.43	1.37	0.37	2.79	3.48	1.95	1.31
${\hat{H}}_{2}/4{\hat{H}}_{1}$	0.23	0.33	0.20	0.20	0.23	0.17	0.23	0.18
${\;\hat{K}}_{D}/{\;\hat{K}}_{R}$	1.29	5.00	0.77	1.27	1.39	4.07	1.61	0.60
${\hat{h}}^{2}/{\hat{H}}_{2}$	0.46	1.71	1.37	0.41	1.64	–0.16	1.46	–0.31
***h²***_***bs***_	0.90	0.67	0.85	0.98	0.64	0.97	0.74	0.94
***h²***_***ns***_	0.64	0.64	0.60	0.87	0.13	0.08	0.16	0.26
${r(\hat{W}}_{r}+{\;\hat{V}}_{r},{\hat{Y}}_{r})$	0.61	–0.65	0.55	0.95	0.94	–0.78	0.92	–0.20
**Components**	**NGS**		**TKW**		**GY**		**BIO**	
	**F**_**1**_	**F**_**2**_	**F**_**1**_	**F**_**2**_	**F**_**1**_	**F**_**2**_	**F**_**1**_	**F**_**2**_
**F**_**1**_: $({\hat{H}}_{1}/\hat{D})$ **F**_**2**_: $(¼{\hat{H}}_{1}/\hat{D})$	11.95	0.73	1.11	1.61	1.48	2.68	3.18	3.81
${\hat{H}}_{2}/4{\hat{H}}_{1}$	0.22	0.20	0.31	0.20	0.21	0.20	0.24	0.23
${\;\hat{K}}_{D}/{\hat{K}}_{R}$	0.64	1.05	0.60	15.35	1.84	13.74	0.61	–0.04
${\hat{h}}^{2}/{\hat{H}}_{2}$	1.44	1.04	2.57	–0.09	1.18	0.35	1.47	0.55
***h²***_***bs***_	0.91	0.96	0.50	0.97	0.84	0.98	0.81	0.95
***h²***_***ns***_	0.26	0.58	0.32	0.41	0.27	0.15	0.31	0.04
***r(***${\hat{W}}_{r}$ +${\;\hat{V}}_{r}$***,*** ${\hat{Y}}_{r}$***)***	–0.16	–0.97	–0.55	0.20	0.95	–0.93	0.92	0.31

PH: Plant height, SL: Spike length, SW: Spike weight, NS: Number of spikes plant^-1^, NGS: Number of grains spike^-1^, TKW: Thousand kernel weight, GY: Grain yield, BIO: Above-ground biomass. $({\hat{H}}_{\text{1}}/\hat{D}$): Dominance means degree in F_1_, $(¼{\hat{H}}_{\text{1}}/\hat{D}$): Dominance means degree in F_2_, ${\hat{H}}_{\text{2}}/\text{4}{\hat{H}}_{\text{1}}$: Allele distribution symmetry, ${\hat{K}}_{D}/{\hat{K}}_{R}$: Relation dominant/recessive, ${\hat{h}}^{\text{2}}/{\hat{H}}_{\text{2}}$: Number of genes with dominance, *h²*_*bs*_: Broad-sense heritability, *h²*_*ns*_: Narrow-sense heritability.

#### 3.3.2. Spike length.

**Fig 2:**
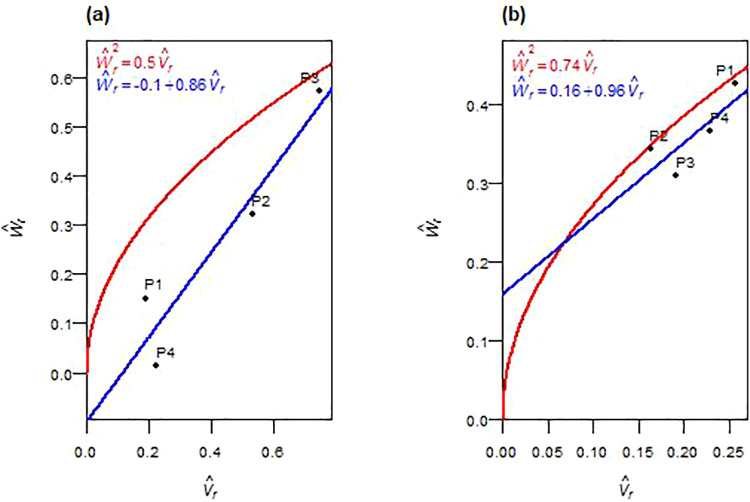
${\hat{W}}_{r}-{\hat{V}}_{r}$ graphs for spike length in F_1_ (a) and F_2_ (b) durum wheat.

Spike length was influenced by both additive and non-additive genetic effects across F_1_ and F_2_ generations (Tables 4, 5). In F_1_, additive variance ($\hat{D}$) and dominance components (${\hat{H}}_{\text{1}}$, ${\hat{H}}_{\text{2}}$ and ${\hat{h}}^{\text{2}}$) were significant. The average degree of dominance exceeded unity ($({\hat{H}}_{\text{1}}/\hat{D})>\text{1}$), and the regression line of the ${\hat{W}}_{r}$– ${\;\hat{V}}_{r}$ graph intercepted below the origin (Fig 2a). In F_2_, the dominance components (${\hat{H}}_{\text{1}}$ and ${\hat{H}}_{\text{2}}$) were non-significant, and deviation (${\hat{h}}^{\text{2}}$) became non-significant, while additive effects remained. The degree of dominance was reduced ($(¼{\hat{H}}_{\text{1}}/\hat{D})<\text{1}$), with the regression line intercepting above the origin (Fig 2b). The ratio ${\hat{H}}_{\text{2}}$*/4*${\hat{H}}_{\text{1}}$ was less than 0.25 in both generations. The sign of $\hat{F}$ and ${\;\hat{K}}_{D}/{\;\;\hat{K}}_{R}$ ratio shifted from negative and < 1 in F_1_ to > 1 in F_2_. The ratio ${\hat{h}}^{\text{2}}/{\hat{H}}_{\text{2}}$ indicated different numbers of dominant gene blocks in F_1_ and F_2_. The correlation ($r({\hat{W}}_{r}+{\;\hat{V}}_{r},{\hat{Y}}_{r})$) was positive in both generations. Parental positions on the ${\hat{W}}_{r}-{\hat{V}}_{r}$ graphs shifted across generations. In F_1_, P1 and P4 had greater concentrations of dominant alleles (closest to the origin), while P3 was richer in recessives (farthest from the origin) (Fig 2a). In F_2_, the overall genetic balance shifted, with P1 acquiring more recessive alleles, and P2 and P3 showing dominance-rich profiles (Fig 2b). Environmental variance ($\hat{E}$) was significant in F_1_. High narrow-sense heritability (*h²*_*ns*_) values were maintained across generations.

#### 3.3.3. Spike weight.

**Fig 3:**
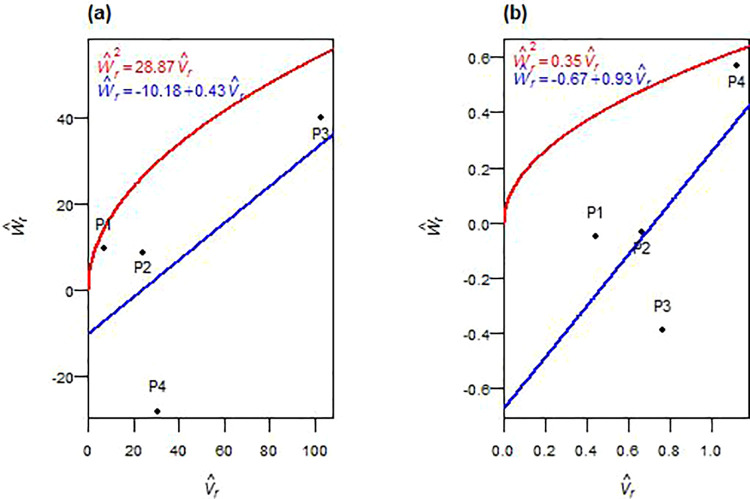
${\hat{W}}_{r}-{\hat{V}}_{r}$ graphs for spike weight in F_1_ (a) and F_2_ (b) durum wheat.

The inheritance of spike weight demonstrated a predominant influence of non-additive genetic effects across both F_1_ and F_2_ generations (Tables 4, 5). In F_1_, most variance components, including $\hat{D}$, ${\hat{H}}_{\text{1}}$, ${\hat{H}}_{\text{2}}$, $\hat{F}$, and $\hat{E}$, were non-significant, whereas the dominance deviation (${\hat{h}}^{\text{2}}$) was significant. In F_2_, dominance components (${\hat{H}}_{\text{1}}$ and ${\hat{H}}_{\text{2}}$) were significant while $\hat{D}$, ${\hat{h}}^{\text{2}}$, $\hat{F}$, and $\hat{E}$ remained non-significant. Overdominance was suggested by average dominance degrees (*√(*${\hat{H}}_{\text{1}}/\hat{D})>\text{1}$ in F_1_ and $(¼{\hat{H}}_{\text{1}}/\hat{D})>\text{1}$ in F_2_) and the position of the regression line on the ${\hat{W}}_{r}$– ${\;\hat{V}}_{r}$ graph, which intercepted below the origin in both generations (Fig 3a, b). The ${\hat{H}}_{\text{2}}/\text{4}{\hat{H}}_{\text{1}}$ ratio was less than 0.25 in both generations. In F_1_, the positive $\hat{F}$ and ${\;\hat{K}}_{D}$*/*${\;\hat{K}}_{R}$> 1 implied a slight excess of dominant alleles, whereas in F_2_, the negative $\hat{F}$ and ${\;\hat{K}}_{D}$/ ${\;\hat{K}}_{R}$< 1 suggested a shift toward recessive alleles. The estimated gene number ratio (${\hat{h}}^{\text{2}}/{\hat{H}}_{\text{2}}$) indicated that one dominance gene or gene block influenced SW in F_1_, whereas in F_2_ it returned a negative value, likely underestimating gene number. The correlation between array point placement and parental mean (*r(*${\hat{W}}_{r}$+ ${\;\hat{V}}_{r}$, ${\;\hat{Y}}_{r}$)) was positive in F_1_ and negative in F_2_. P3 was farthest from the origin, carrying the most recessive alleles, whereas P4 and P1, being closest to the origin, contained dominant alleles in F_1_ (Fig 3a). In F_2_, P1 had the maximum dominant alleles, and P4 contained the highest number of dominant alleles (Fig 3b). Broad-sense heritability (*h²*_*bs*_) was high in both generations. In contrast, narrow-sense heritability (*h²*_*ns*_) remained low.

#### 3.3.4. Number of spikes per plant.

**Fig 4:**
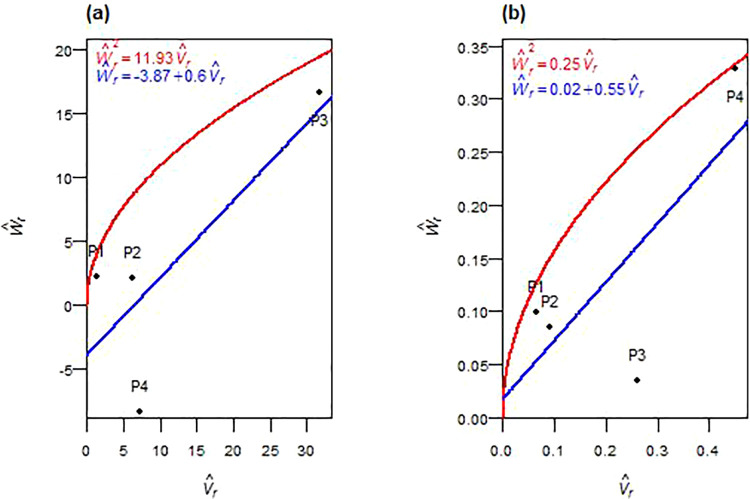
${\hat{W}}_{r}-{\hat{V}}_{r}$ graphs for the number of spikes plant^-1^ in F_1_ (a) and F_2_ (b) durum wheat.

The number of spikes per plant exhibited non-additive genetic control across both F_1_ and F_2_ generations (Tables 4, 5). In F_1_, all primary genetic and environmental components ($\hat{D}$, ${\hat{H}}_{\text{1}}$, ${\hat{H}}_{\text{2}}$, $\hat{F}$, $\hat{E}$) were non-significant, except for the dominance deviation (${\hat{h}}^{\text{2}}$), which was significant. In F_2_, both additive ($\hat{D}$) and dominance (${\hat{H}}_{\text{1}}$) variances were significant, whereas ${\hat{H}}_{\text{2}}$, ${\hat{h}}^{\text{2}}$, $\hat{F}$, and $\hat{E}$ remained non-significant. In both generations, overdominance was evident, as the average degree of dominance exceeded unity: *√(*${\hat{H}}_{\text{1}}$*/*$\hat{D}$) > 1 in F_1_ and $\left(¼{\hat{H}}_{\text{1}}/\hat{D}\right)$ > 1 in F_2_. The regression of ${\hat{W}}_{r}$ on ${\;\hat{V}}_{r}$ supported these observations: the regression line intercepted the axis below the origin in F_1_ and slightly above the origin in F_2_ (Fig 4a, b). The frequency ratio of alleles (${\hat{H}}_{\text{2}}/\text{4}{\hat{H}}_{\text{1}}$) deviated from 0.25 in both generations. In F_1_, $\hat{F}$ was positive and ${\;\hat{K}}_{D}$*/*${\;\hat{K}}_{R}$> 1, while in F_2_, the $\hat{F}$ was negative and ${\hat{K}}_{D}$*/*${\hat{K}}_{R}$ < 1. NS had around one gene or gene block in F_1_, as shown by ${\hat{h}}^{\text{2}}/{\hat{H}}_{\text{2}}$, with a negative value in F_2_. The correlation *r(*${\hat{W}}_{r}$+  ${\hat{V}}_{r}$,   ${\hat{Y}}_{r}$) was strongly positive in F_1_ and weakly negative in F_2_. Parent positioning along the regression line indicated that P3 was farthest, with a higher concentration of recessive alleles, and P1 was closest to the origin, with a predominance of dominant genes in F_1_ (Fig 4a). In F_2_, P1 and P2 retained the highest concentration of dominant alleles, while P4 exhibited the most recessive ones (Fig 4b). Broad-sense heritability (*h²*_*bs*_) was high, whereas narrow-sense heritability (*h²*_*ns*_) was low in both generations.

#### 3.3.5. Number of grains per spike.

**Fig 5:**
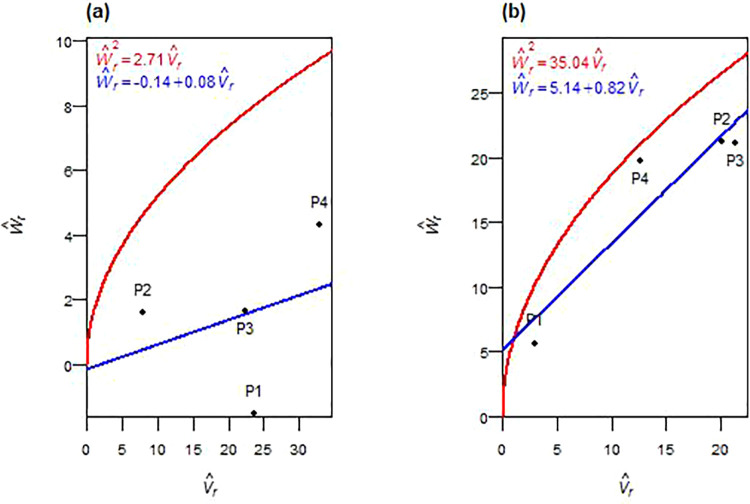
${\hat{W}}_{r}-{\hat{V}}_{r}$ graphs for the number of grains spike^-1^ in F_1_ (a) and F_2_ (b) durum wheat.

The number of grains per spike was influenced by non-additive gene action in both F_1_ and F_2_ generations. In F_1_, dominance components (${\hat{H}}_{\text{1}}$, ${\hat{H}}_{\text{2}}$, and ${\hat{h}}^{\text{2}}$) were significant, while the additive variance ($\hat{D}$) was not. In F_2_, both additive and dominance components ($\hat{D}$, ${\hat{H}}_{\text{1}}$, ${\hat{H}}_{\text{2}}$ and ${\hat{h}}^{\text{2}}$) were significant. The degree of dominance exceeded unity in F_1_ $(\sqrt{\frac{{\hat{H}}_{\text{1}}}{\hat{D}}}>\text{1}$) and was less than unity in F_2_ ($(¼{\hat{H}}_{\text{1}}/\hat{D})<\text{1}$). The regression of ${\hat{W}}_{r}$ on ${\hat{V}}_{r}$ is intercepted below the origin in F_1_ and slightly above it in F_2_ (Fig 5a, b). Allelic frequency ratios (${\hat{H}}_{\text{2}}/\text{4}{\hat{H}}_{\text{1}}$) deviated from 0.25. The sign of $\hat{F}$ shifted from negative in F_1_ to positive in F_2_, and ${\hat{K}}_{D}/{\hat{K}}_{R}$ increased across generations. The gene ratio (${\hat{h}}^{\text{2}}/{\hat{H}}_{\text{2}}$) indicated a dominant gene/gene block in both generations. The correlation $r({\hat{W}}_{r},{\;\hat{V}}_{r},{\hat{Y}}_{r})$ was weakly negative in F_1_ and strongly negative in F_2_. Parent P3 (farthest from origin) had more recessive alleles in F_1_, while P2 (closest to origin) was more dominant (Fig 5a). In F_2_, P1 had more dominant alleles, whereas P2 and P3 had more recessive ones (Fig 5b). Broad-sense heritability (*h²*_*bs*_) was high across both generations. Narrow-sense heritability (*h²*_*ns*_) was low in F_1_ but moderate in F_2_.

#### 3.3.6. Thousand kernel weight.

**Fig 6:**
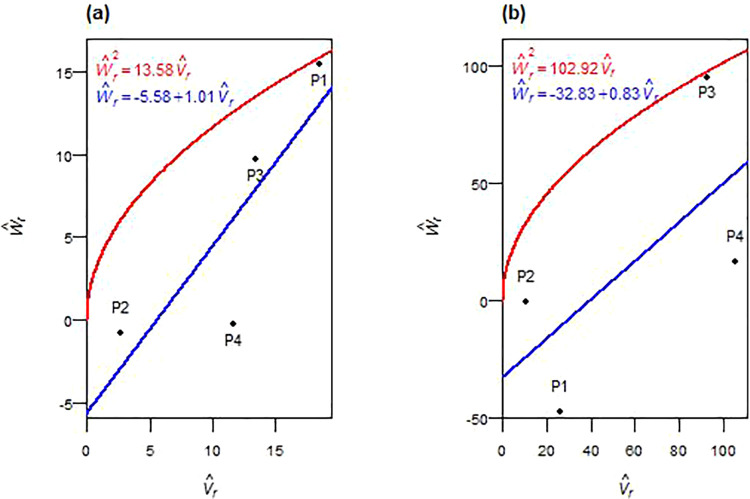
${\hat{W}}_{r}-{\hat{V}}_{r}$ graphs for thousand kernel weight in F_1_ (a) and F_2_ (b) durum wheat.

In both F_1_ and F_2_ generations, the additive genetic variance ($\hat{D}$) was statistically significant (Tables 4, 5). In F_1_, dominance variance components (${\hat{H}}_{\text{1}}$ and ${\hat{H}}_{\text{2}}$) were not significant but had higher magnitudes than $\hat{D}$. In F_2_, both ${\hat{H}}_{\text{1}}$ and ${\hat{H}}_{\text{2}}$ became statistically significant and remained larger than $\hat{D}$. The dominance deviation (${\hat{h}}^{\text{2}}$) was significant in both generations. The average degree of dominance was greater than one in both generations: *√(*${\hat{H}}_{\text{1}}$/  $\hat{D}$) > 1 in F_1_ and $\left(¼{\hat{H}}_{\text{1}}/\hat{D}\right)$ > 1 in F_2_. Graphical analysis (Fig 6a, b) showed that the regression line of ${\hat{W}}_{r}$ on ${\;\hat{V}}_{r}$ intercepted the ${\hat{W}}_{r}$-axis below the origin in both generations. The array distribution in F_1_ positioned P2 nearest to the origin, suggesting more dominant alleles, while P1 was farthest, indicating a higher proportion of recessive alleles (Fig 6a). In F_2_, P2 remained close to the origin, with P3 farther out (Fig 6b). The allele symmetry ratio (${\hat{H}}_{\text{2}}/\text{4}{\hat{H}}_{\text{1}}$) deviated from 0.25 in both generations, and the covariance component ($\hat{F}$) changed from negative in F_1_ to strongly positive in F_2_. The dominant-to-recessive allelic ratio (${\hat{K}}_{D}$*/*${\;\hat{K}}_{R}$) increased in F_2_ relative to F_1_. The ratio ${\hat{h}}^{\text{2}}/{\hat{H}}_{\text{2}}$ indicated that multiple genes or gene blocks with dominance effects contributed to TKW expression in the F_1_ generation. In F_2_, no dominant genes or gene blocks were observed. The correlation *r(*${\hat{W}}_{r}$ + ${\;\hat{V}}_{r}$,  ${\;\hat{Y}}_{r}$) was moderately negative in F_1_ and became weakly positive in F_2_. Broad-sense heritability (*h²*_*bs*_) was high in both generations, while narrow-sense heritability (*h²*_*ns*_) was moderate, with a slight increase from F_1_ to F_2_.

#### 3.3.7. Grain yield.

**Fig 7:**
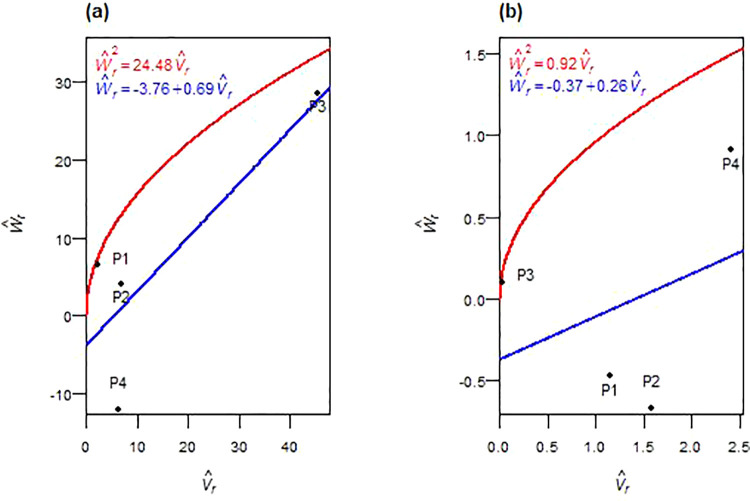
${\hat{W}}_{r}-{\hat{V}}_{r}$ graphs for grain yield in F_1_ (a) and F_2_ (b) durum wheat.

The genetic basis of grain yield revealed distinct trends in both F_1_ and F_2_ generations, with varying contributions from additive and non-additive effects (Tables 4, 5). In the F_1_ generation, the additive genetic variance ($\hat{D}$) was significant, while dominance components (${\hat{H}}_{\text{1}}$ and ${\hat{H}}_{\text{2}}$) were not significant. In F_2_, the additive component ($\hat{D}$) was not significant, but both dominance variance components (${\hat{H}}_{\text{1}}$ and ${\hat{H}}_{\text{2}}$) became significant. Dominance deviation (${\hat{h}}^{\text{2}}$) was significant in F_1_ and non-significant in F_2_. The average degree of dominance exceeded unity in both generations: $({\hat{H}}_{\text{1}}/\hat{D})>\text{1}$ in F_1_ and $(¼{\hat{H}}_{\text{1}}/\hat{D})>\text{1}$ in F_2_. The regression of ${\hat{W}}_{r}$ on ${\;\hat{V}}_{r}$ intersected the ${\hat{W}}_{r}$-axis below the origin in both generations (Fig 7a, b). The ${\hat{H}}_{\text{2}}/\text{4}{\hat{H}}_{\text{1}}$ ratio deviated from 0.25, and the $\hat{F}$ component was positive in both generations. The ${\;\hat{K}}_{D}$/ ${\;\hat{K}}_{R}$ ratio exceeded unity in F_1_ and F_2_. The ${\hat{h}}^{\text{2}}/{\hat{H}}_{\text{2}}$ ratio suggested a dominant gene or gene block contributed in both generations. The correlation coefficient $r({\hat{W}}_{r}+{\hat{V}}_{r},{\hat{Y}}_{r})$ was positive in F_1_ and negative in F_2_. In F_1_, P3 was situated farther from the origin, while parents P1 and P2 were closer (Fig 7a). In F_2_, P3 shifted closer to the origin, and P4 was positioned farther (Fig 7b). Broad-sense heritability (*h²*_*bs*_) was high across generations, whereas narrow-sense heritability (*h²*_*ns*_) was low.

#### 3.3.8. Above-ground biomass.

**Fig 8:**
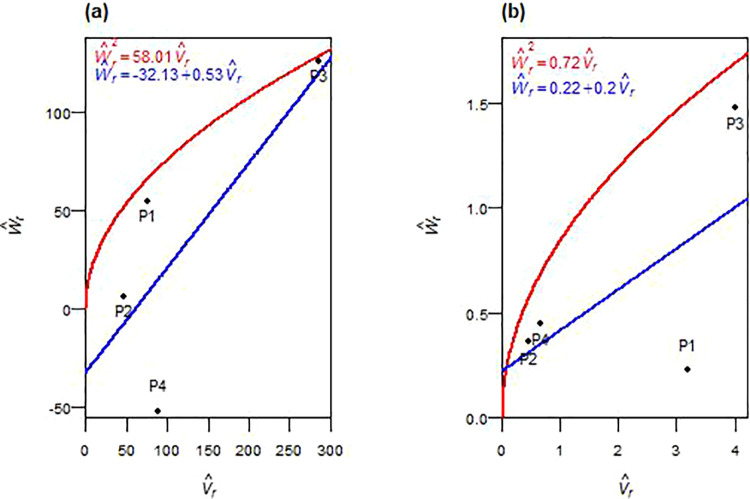
${\hat{W}}_{r}-{\hat{V}}_{r}$ graphs for above-ground biomass in F_1_ (a) and F_2_ (b) durum wheat.

The genetic analysis of above-ground biomass revealed that non-additive effects played a predominant role in both F_1_ and F_2_ generations (Tables 4, 5). In both cases, the additive variance component ($\hat{D}$) was consistently lower than the dominance-related components (${\hat{H}}_{\text{1}}$, ${\hat{H}}_{\text{2}}$, and ${\hat{h}}^{\text{2}}$). Among these, only the dominance deviation (${\hat{h}}^{\text{2}}$) reached significance in F_1_. The dominance components (${\hat{H}}_{\text{1}}$ and ${\hat{H}}_{\text{2}}$) had larger magnitudes than $\hat{D}$ across both generations, though statistical significance was not achieved in most cases. The calculated average degree of dominance, as estimated by $\left({\hat{H}}_{\text{1}}/\hat{D}\right)$ in F_1_ and $\left(¼{\hat{H}}_{\text{1}}/\hat{D}\right)$ in F_2_, exceeded unity. The regression of ${\hat{W}}_{r}$ on ${\;\hat{V}}_{r}$ intersected below the origin in F_1_ and above the origin but within the lower half of the distribution in F_2_ (Fig 8a, b). The ${\hat{H}}_{\text{2}}/\text{4}{\hat{H}}_{\text{1}}$ ratio deviated from 0.25 in both generations. Negative $\hat{F}$ values and ${\;\hat{K}}_{D}$*/*${\;\hat{K}}_{R}$ ratios below one were observed in F_1_ and F_2_. The ratio ${\hat{h}}^{\text{2}}/{\hat{H}}_{\text{2}}$ indicated that one gene or gene block was involved in biomass inheritance in F_1_ and F_2_. The correlation *r(*${\hat{W}}_{r}$+ ${\;\hat{V}}_{r}$,  ${\;\hat{Y}}_{r}$) was strongly positive in F_1_ and moderately positive in F_2_. Parental positions along the ${\hat{W}}_{r}$*–*${\;\hat{V}}_{r}$ regression line varied: P3 was near the recessive end in both generations, P2 toward the dominant end, and P1 and P4 in intermediate positions (Fig 8a, b). Broad-sense heritability (*h²*_*bs*_) was high in both generations, while narrow-sense heritability (*h²*_*ns*_) was moderate to low.

## 4. Discussion

This study employed Hayman’s diallel analysis across F_1_ and F_2_ generations to dissect the genetic architecture of eight yield-related traits in durum wheat under semi-arid Mediterranean conditions. Systematic F_1_–F_2_ comparisons reveal dynamic shifts in gene action, distinguishing traits with stable genetic architectures suitable for early selection from those with generation-dependent patterns that require delayed or alternative strategies. This comparative framework is particularly valuable under Algerian semi-arid conditions, where environmental stress can significantly alter gene expression and breeding outcomes, providing climate-relevant insights for Mediterranean wheat improvement. The generation-specific patterns observed enable the development of a trait-specific breeding decision matrix integrating genetic architecture, heritability, and parent-specific allelic contributions to guide optimal selection timing and methodology under semi-arid stress.

The significant genotypic differences across both generations confirm substantial genetic variability available for selection. This aligns with previous diallel studies in wheat that have reported considerable diversity for grain yield and its components in segregating populations, including significant genetic variation among both F_1_ hybrids and their derived F_2_ generations [17–21]. The exceptional variation in traits such as spike weight and biomass among progenies, despite non-significant parental differences, indicates strong non-additive genetic effects and transgressive segregation, patterns that critically inform breeding strategy selection. The reduction in significance for plant height from F_1_ to F_2_ reflects the breakdown of heterotic combinations through recombination, a typical consequence of segregation that underscores the dynamic nature of gene expression across generations and reinforces the importance of evaluating multiple generations to optimize breeding strategies.

The additive-dominance model showed generation-dependent adequacy across traits. In F_1_, the model was generally adequate for plant height, spike length, number of spikes, grain yield, and biomass. In contrast, partial adequacy for spike weight, grain number per spike, and thousand-kernel weight suggested potential epistatic interactions or environmental effects [7,8]. Model adequacy improved in F_2_ for the latter three traits due to stabilization of segregating alleles, whereas minor deviations persisted for plant height, spike length, grain yield, and biomass. Despite partial adequacy for some traits, the variance component estimates remain highly valuable for distinguishing additive versus dominance variance, the fundamental basis for breeding strategy selection, as demonstrated in numerous diallel studies [22–28]. This generation-specific pattern of model adequacy, combined with variance component analysis, provides a solid foundation for trait-specific breeding recommendations.

Plant height demonstrated stable additive inheritance across generations, making it a highly reliable trait for early-generation selection. The predominance of additive variance with minimal dominance, commonly observed in advanced generations [29], and partial dominance align with previous wheat studies [17–19]. Unidirectional dominance in F_2_, with higher proportions of dominant alleles in both generations, and asymmetric allele distribution indicated unequal parental contributions reflecting directional allele segregation. The genetic parameters suggested at least one major gene or gene block regulating plant height, consistent with earlier reports on significant loci influencing this trait [22,30]. The shift in correlation between (${\hat{W}}_{r}$+ ${\;\hat{V}}_{r}$) and ${\;\hat{Y}}_{r}$—from positive in F_1_ to negative in F_2_, indicates a transition from P2-associated dominant height-reducing alleles to P3-associated recessive alleles becoming influential, likely due to diminished dominance masking. High broad- and narrow-sense heritability confirm strong genetic control and enable confident selection for optimal plant stature in early segregating populations, consistent with earlier reports [20,21,31,32]. These findings support pedigree selection in F_2_–F₃ generations, with P3 as a valuable parent for optimal plant stature under semi-arid conditions.

Spike length exhibited a distinct generational shift from dominance-driven inheritance in F_1_ to additive-dominated inheritance in F_2_. The initial overdominance reflects strong heterotic effects arising from favorable heterozygous combinations, consistent with findings in other wheat populations [18,23,31,33]. By F_2_, dominance diminished, while partial dominance prevailed, suggesting recombination-mediated stabilization of additive effects, a typical outcome in self-pollinating populations [13]. Genetic analysis suggested the influence of at least one gene or gene block in F_1_, with effects diminishing as segregation progressed [23,33]. Notably, the persistent positive correlation *r*(${\hat{W}}_{r}$+ ${\hat{V}}_{r}$, ${\hat{Y}}_{r}$) across generations identified recessive alleles as trait-enhancing (P3-rich in F_1_; P2- and P3-rich in F_2_), valuable for breeding programs aiming to accumulate favorable recessive alleles through recurrent selection [30,34]. Although environmental variance was significant in F_1_, high narrow-sense heritability in both generations confirmed reliable allele transmission, supporting pedigree selection from F₃ onward, especially when leveraging P3 as a donor parent for favorable recessive alleles.

Spikes’ weight, number of spikes, and biomass exhibited persistent non-additive control requiring alternative strategies. For spike weight, overdominance persisted across both generations, possibly reflecting dominant-by-dominant interactions in F_2_. Similar heterotic effects for biomass and yield traits have been reported in durum and bread wheat [22,35]. Asymmetric allelic distribution and an increased frequency of the recessive allele, consistent with recombination effects [29], indicated unequal parental contributions, a common feature of diallel crosses involving genetically diverse parents [23]. The genetic architecture indicated a single primary dominance-influencing locus in F_1_, though a reduction in F_2_ may reflect epistatic interactions or unequal gene effects [36–38]. The correlation between (${\hat{W}}_{r}$+ ${\hat{V}}_{r}$) and ${\hat{Y}}_{r}$ shifted dramatically from strongly positive in F_1_ (P3-rich recessive) to strongly negative in F_2_ (P1-rich dominant), indicating complex recombination-driven reconfigurations. The number of spikes followed similar non-additive patterns, consistent with diallel studies [23,32,39], with overdominance and heterotic gene action in F_1_. By F_2_, recombination reshaped the genetic architecture, with continued non-additive effects alongside emerging small additive contributions. The genetic parameters implied the presence of at least one major dominant gene in F_1_, with a reduction in F_2_ potentially reflecting epistatic interactions [36,37]. The correlation *r(*${\hat{W}}_{r}$+ ${\hat{V}}_{r}$,  ${\hat{Y}}_{r}$) shifted from strongly positive in F_1_ (P3-rich recessive) to weakly negative in F_2_ (P1- and P2-rich dominant), suggesting non-allelic interactions [38] or loss of favorable recessive combinations. Biomass showed comparable inheritance patterns, with dominance exceeding additive variance, consistent with reports of complex non-additive interactions [18,22,39]. Overdominance was evident, especially in early generations, supporting heterotic contributions. The genetic parameters implied control by a single dominant locus or tightly linked gene blocks, consistent with Fellahi et al. [22] and El-Gammaal and Yahya [40]. The positive correlations *r(*${\hat{W}}_{r}$+ ${\hat{V}}_{r}$,  ${\hat{Y}}_{r}$), strong in F_1_ and moderate in F_2_, indicated that P3-rich recessive alleles significantly influenced biomass, though effectiveness diminished with segregation. Despite high broad-sense heritability confirming strong genetic control for all three traits, consistently low narrow-sense heritability indicated minimal additive influence. These findings support delayed selection (F₄–F₅) when additive variance becomes more accessible [41,42], or hybrid breeding to exploit heterosis with P1 (spike weight), P1 and P2 (number of spikes), and P3 (biomass) as key parents.

Grain number per spike and thousand-kernel weight showed mixed inheritance, with generation-specific patterns. For grain number per spike, the genetic architecture transitioned from net dominance in F_1_ to increasing additive contributions in F_2_, consistent with previous wheat diallel studies [23,43]. Overdominance in F_1_ shifted to partial dominance in F_2_, as evidenced by improved narrow-sense heritability, a pattern consistent with mixed inheritance models [44]. The genetic parameters implied one dominant gene, or gene blocks consistent with polygenic trait behavior [23], while the correlation between (${\hat{W}}_{r}$+ ${\hat{V}}_{r}$) and ${\hat{Y}}_{r}$ shifted from weak in F_1_ to strongly negative in F_2_, confirming P1’s transition to dominant allele predominance [32,40] and establishing P1’s specific breeding value. Rising narrow-sense heritability in F_2_ indicated greater accessibility of additive variance for thousand kernel weight; both additive and non-additive effects governed inheritance, with dominance playing a more prominent role. Additive variance in both generations indicated selection potential, though persistent overdominance revealed strong non-additive contributions, frequently reported for spike grain weight in wheat [22,23,33]. The gene ratio indicated at least two dominant genes or gene blocks in F_1_, aligning with Chaudhari et al. [23], and approached zero in F_2_, likely due to recombination breaking gene clusters [36–38]. The correlation ${r(\hat{W}}_{r}+{\hat{V}}_{r},{\hat{Y}}_{r})$ shifted from moderately negative in F_1_ to weakly positive in F_2_, implying that P2-rich dominant alleles predominantly increased grain size in F_1_, while P3-rich recessive alleles increased this trait in F_2_. While high broad-sense heritability reflected strong genetic control, moderate narrow-sense heritability suggested limited early-generation efficiency. These findings support selection in F₃–F₄ generations for grain number per spike with P1 as key parent, while thousand kernel weight requires a combined strategy of F₄–F₅ selection or hybrid breeding using P2 (F_1_-favorable) and P3 (F_2_-favorable).

Grain yield exhibited exceptional complexity requiring population-level approaches. Significant additive effects in F_1_ but not in F_2_ suggested recombination disrupted favorable additive combinations, while dominance variance became significant in F_2_, emphasizing the non-additive influence observed in previous studies [30,40,45]. Significant dominance deviation in F_1_ indicated unidirectional dominance with multiple positive genes, though absence in F_2_ may reflect cancellation of dominance effects [46]. Persistent overdominance across generations aligns with heterosis-driven yield enhancement [17,32,34,43]. Asymmetric allele distribution reinforced the dominance of the dominant allele, supporting the potential for heterosis. The genetic parameters implied that a single gene or gene block controls yield, consistent with Chaudhari et al. [23] and Afridi et al. [47]. The correlation *r(*${\hat{W}}_{r}$+ ${\hat{V}}_{r}$,  ${\hat{Y}}_{r}$) shifted dramatically from strongly positive in F_1_, where P3-rich recessive alleles enhanced yield, to strongly negative in F_2_, where P3-rich dominant alleles increased yield, highlighting P3’s transition and confirming exceptional breeding value for hybrid development [18,19]. Despite high broad-sense heritability reflecting strong genetic control, low narrow-sense heritability in both generations underscored non-additive dominance limiting early-generation selection efficiency, corroborating reports of low to moderate heritability for grain yield in wheat [32,39]. These findings support the breeding recommendation that grain yield improvement under semi-arid conditions should prioritize recurrent selection or hybrid development to capitalize on non-additive genetic effects, with P3 serving as a cornerstone parent for both heterosis exploitation and population improvement. Bulk breeding methods may also prove effective, as suggested by Acquaah [13].

While this study provides a comprehensive biometrical framework for selecting breeding strategies, integrating molecular approaches could significantly enhance its applicability. The genetic components, particularly for traits governed by a few significant genes or gene blocks (plant height, spike length), offer candidates for QTL (Quantitative Trait Locus) mapping [48]. Consistent additive control of plant height suggests stable, fixable loci suitable for marker-assisted selection [49]. Identification of parents with favorable alleles (P1 for grain number per spike, P3 for spike length, and biomass) provides targeted resources for association mapping or genomic selection [50]. Validating biometrical estimates with genomic tools would confirm loci involved and dissect epistatic interactions suggested by partial model adequacy. Ultimately, combining phenotypic-based parameters with genomic data could accelerate cultivar development for semi-arid Mediterranean regions.

## 5. Conclusion

This diallel analysis provided key insights into the genetic architecture of yield-related traits in durum wheat across F_1_ and F_2_ generations. Plant height was predominantly under additive gene control across both generations, reflecting stable inheritance conducive to effective selection. In contrast, spike length and grains per spike shifted from overdominance in F_1_ to partial dominance in F_2_, while yield components essentially exhibited non-additive effects, with marked overdominance. The significant heterozygous dominance effects (${\hat{h}}^{\text{2}}$) detected in F_1_ point to unidirectional dominance and strong heterotic responses for most traits. This pattern was not retained in F_2_, suggesting that dominance observed in F_1_ was primarily attributable to heterozygosity, which was reduced in F_2_ due to increased homozygosity. Allele distribution among parents was uneven, with specific genotypes contributing key alleles: P3 provided beneficial recessive alleles for spike traits in F_1_, while P1 contributed dominant alleles improving grain traits in F_2_. Shifts in the correlations between (${\hat{W}}_{r}$+ ${\hat{V}}_{r}$) and ${\hat{Y}}_{r}$ supported changes in dominance patterns across generations, as observed in traits such as spike weight and grain yield, where recessive alleles enhanced trait expression in the F_1_ generation.

In contrast, the dominant alleles had a more substantial effect in the F_2_ generation. Most traits appeared to be governed by a major gene or a tightly linked gene block. High broad-sense heritability confirmed strong genetic control, while moderate-to-low narrow-sense heritability for yield components indicated limited early-generation selection efficiency. These findings emphasize the need for trait-specific and generation-appropriate breeding strategies. Conventional selection is suitable for traits with additive effects, whereas complex, non-additive traits will benefit from recurrent selection or delayed selection in later generations to maximize genetic gain.

## Supporting information

S1 File Supplementary data. (DOCX)
