# Supplementary material for: Hayman’s diallel analysis for yield-related traits in F1 and F2 durum wheat (Triticum durum Desf.) progenies
Source: PLoS One. 2026 Feb 24;21(2):e0342977. doi: 10.1371/journal.pone.0342977 (PMC12931811; doi:10.1371/journal.pone.0342977)
Supplement: S1 File — (DOCX) [file pone.0342977.s001.docx]

Table S1Raw experimental data for F₁ durum wheat diallel progenies.

| Female parent | Male parent | Block | PH | SL | SW | NS | NGS | TKW | GY | BIO |
| --- | --- | --- | --- | --- | --- | --- | --- | --- | --- | --- |
| P1 | P1 | 1 | 110.75 | 8.75 | 14.25 | 8.00 | 52.80 | 21.78 | 6.65 | 42.65 |
| P1 | P2 | 1 | 73.50 | 8.62 | 16.13 | 9.00 | 40.35 | 28.17 | 9.28 | 31.30 |
| P1 | P3 | 1 | 80.80 | 7.70 | 16.38 | 8.00 | 44.28 | 32.37 | 9.88 | 32.20 |
| P1 | P4 | 1 | 82.63 | 7.50 | 12.53 | 7.00 | 34.65 | 29.16 | 6.79 | 29.48 |
| P2 | P2 | 1 | 66.00 | 8.28 | 22.85 | 10.50 | 50.70 | 28.49 | 13.13 | 39.00 |
| P2 | P3 | 1 | 68.98 | 7.41 | 15.32 | 7.50 | 45.53 | 30.47 | 9.06 | 28.67 |
| P2 | P4 | 1 | 64.00 | 6.56 | 9.70 | 6.71 | 34.75 | 26.91 | 5.90 | 18.70 |
| P3 | P3 | 1 | 66.00 | 8.91 | 32.65 | 16.50 | 49.70 | 29.43 | 18.45 | 59.40 |
| P3 | P4 | 1 | 58.00 | 6.33 | 7.00 | 3.00 | 35.67 | 40.74 | 4.43 | 15.20 |
| P4 | P4 | 1 | 88.67 | 7.21 | 17.40 | 8.33 | 48.20 | 29.77 | 10.27 | 39.73 |
| P1 | P1 | 2 | 106.00 | 9.19 | 17.50 | 9.00 | 47.90 | 22.34 | 8.50 | 46.50 |
| P1 | P2 | 2 | 73.63 | 8.41 | 14.88 | 7.75 | 44.68 | 29.50 | 8.96 | 26.93 |
| P1 | P3 | 2 | 85.00 | 8.44 | 23.46 | 11.60 | 45.48 | 27.30 | 12.77 | 47.14 |
| P1 | P4 | 2 | 86.88 | 7.87 | 15.25 | 7.25 | 35.55 | 36.41 | 8.69 | 32.28 |
| P2 | P2 | 2 | 62.50 | 7.97 | 19.40 | 11.50 | 49.30 | 25.03 | 10.91 | 32.90 |
| P2 | P3 | 2 | 68.98 | 7.41 | 15.32 | 7.50 | 45.53 | 30.47 | 9.06 | 28.67 |
| P2 | P4 | 2 | 71.43 | 6.78 | 11.20 | 6.14 | 40.67 | 30.78 | 7.19 | 20.31 |
| P3 | P3 | 2 | 60.50 | 9.03 | 21.35 | 12.50 | 49.70 | 24.61 | 19.24 | 37.00 |
| P3 | P4 | 2 | 44.00 | 7.20 | 2.30 | 1.00 | 41.00 | 32.15 | 1.38 | 5.70 |
| P4 | P4 | 2 | 92.17 | 7.43 | 24.97 | 10.33 | 50.73 | 33.26 | 8.44 | 60.00 |
| P1 | P1 | 3 | 93.50 | 8.26 | 21.45 | 10.50 | 44.70 | 24.87 | 12.48 | 54.30 |
| P1 | P2 | 3 | 77.20 | 8.68 | 14.74 | 6.00 | 45.85 | 30.38 | 7.58 | 24.60 |
| P1 | P3 | 3 | 82.67 | 7.88 | 20.28 | 8.67 | 45.01 | 29.17 | 11.39 | 39.90 |
| P1 | P4 | 3 | 86.83 | 8.12 | 14.03 | 6.67 | 37.27 | 34.58 | 8.06 | 30.73 |
| P2 | P2 | 3 | 66.83 | 7.96 | 27.90 | 15.00 | 49.27 | 26.47 | 16.00 | 43.17 |
| P2 | P3 | 3 | 68.98 | 7.41 | 15.32 | 7.50 | 45.53 | 30.47 | 9.06 | 28.67 |
| P2 | P4 | 3 | 75.17 | 7.38 | 14.37 | 8.17 | 45.38 | 27.01 | 8.64 | 26.77 |
| P3 | P3 | 3 | 63.00 | 8.46 | 33.10 | 18.00 | 50.00 | 30.26 | 20.02 | 56.80 |
| P3 | P4 | 3 | 51.00 | 6.77 | 4.65 | 2.00 | 38.33 | 36.44 | 2.90 | 10.45 |
| P4 | P4 | 3 | 81.75 | 7.21 | 11.25 | 5.25 | 47.66 | 32.71 | 6.60 | 27.30 |

Table S2 Raw experimental data for F₂ durum wheat diallel progenies

| Female parent | Male parent | Block | PH | SL | SW | NS | NGS | TKW | GY | BIO |
| --- | --- | --- | --- | --- | --- | --- | --- | --- | --- | --- |
| P1 | P1 | 1 | 76.80 | 7.30 | 5.90 | 2.90 | 38.60 | 36.80 | 3.90 | 14.20 |
| P1 | P2 | 1 | 72.00 | 6.70 | 4.90 | 3.20 | 40.30 | 40.10 | 3.30 | 15.00 |
| P1 | P3 | 1 | 72.20 | 6.40 | 7.40 | 3.60 | 35.90 | 32.10 | 4.90 | 18.30 |
| P1 | P4 | 1 | 74.70 | 6.70 | 6.00 | 3.50 | 35.40 | 45.60 | 6.70 | 15.80 |
| P2 | P2 | 1 | 58.50 | 5.80 | 7.30 | 3.90 | 30.20 | 40.10 | 5.20 | 14.00 |
| P2 | P3 | 1 | 63.65 | 5.75 | 5.80 | 3.40 | 32.60 | 42.10 | 4.70 | 13.70 |
| P2 | P4 | 1 | 93.70 | 5.90 | 7.40 | 3.80 | 32.40 | 44.30 | 6.30 | 14.30 |
| P3 | P3 | 1 | 56.40 | 5.70 | 6.40 | 3.60 | 26.00 | 48.20 | 4.80 | 12.10 |
| P3 | P4 | 1 | 57.60 | 5.20 | 7.90 | 4.40 | 28.30 | 19.00 | 4.80 | 13.30 |
| P4 | P4 | 1 | 65.30 | 6.10 | 5.50 | 3.00 | 35.90 | 30.90 | 2.80 | 13.90 |
| P1 | P1 | 2 | 81.50 | 7.60 | 6.30 | 2.90 | 40.40 | 37.70 | 4.00 | 15.70 |
| P1 | P2 | 2 | 68.90 | 6.30 | 4.20 | 2.50 | 35.90 | 33.00 | 3.00 | 13.60 |
| P1 | P3 | 2 | 72.90 | 6.60 | 6.50 | 3.90 | 36.30 | 38.70 | 6.10 | 17.30 |
| P1 | P4 | 2 | 73.80 | 6.40 | 5.70 | 2.90 | 34.50 | 50.10 | 5.50 | 16.40 |
| P2 | P2 | 2 | 52.40 | 5.50 | 6.60 | 2.80 | 26.90 | 41.50 | 3.10 | 12.70 |
| P2 | P3 | 2 | 63.65 | 5.75 | 5.80 | 3.40 | 32.60 | 42.10 | 4.70 | 13.70 |
| P2 | P4 | 2 | 59.80 | 5.50 | 6.30 | 2.80 | 29.80 | 34.90 | 6.30 | 14.30 |
| P3 | P3 | 2 | 58.70 | 5.70 | 7.40 | 4.30 | 26.50 | 48.60 | 5.50 | 14.90 |
| P3 | P4 | 2 | 67.70 | 5.60 | 8.70 | 4.80 | 29.90 | 33.20 | 5.40 | 16.40 |
| P4 | P4 | 2 | 57.20 | 5.90 | 5.70 | 3.50 | 30.00 | 21.40 | 2.90 | 12.60 |
| P1 | P1 | 3 | 78.60 | 7.60 | 5.60 | 2.70 | 38.00 | 40.80 | 4.00 | 14.40 |
| P1 | P2 | 3 | 70.00 | 6.50 | 5.60 | 2.70 | 41.50 | 32.70 | 3.70 | 12.30 |
| P1 | P3 | 3 | 65.50 | 6.20 | 5.60 | 2.70 | 34.80 | 34.70 | 3.60 | 17.90 |
| P1 | P4 | 3 | 79.90 | 6.40 | 6.30 | 2.60 | 40.60 | 42.40 | 5.20 | 15.30 |
| P2 | P2 | 3 | 58.50 | 5.70 | 5.80 | 3.40 | 27.90 | 39.40 | 4.30 | 11.40 |
| P2 | P3 | 3 | 63.65 | 5.75 | 5.80 | 3.40 | 32.60 | 42.10 | 4.70 | 13.70 |
| P2 | P4 | 3 | 59.30 | 5.90 | 6.30 | 3.70 | 35.70 | 47.10 | 6.30 | 14.30 |
| P3 | P3 | 3 | 54.00 | 5.60 | 6.30 | 4.00 | 23.20 | 53.80 | 5.10 | 13.50 |
| P3 | P4 | 3 | 62.70 | 5.30 | 7.10 | 4.10 | 26.20 | 30.60 | 4.30 | 14.20 |
| P4 | P4 | 3 | 52.10 | 6.20 | 5.20 | 2.60 | 30.60 | 24.20 | 2.80 | 15.30 |

Table S33 *V̂ᵣ* and *Ŵᵣ* values used to generate Figures 1–8.

| Generation | Traits | Parent | V̂ᵣ | Ŵᵣ |
| --- | --- | --- | --- | --- |
| F_1_ | PH | P1 | 146.08 | 208.30 |
| F_1_ | PH | P2 | 15.87 | 67.75 |
| F_1_ | PH | P3 | 174.61 | 94.87 |
| F_1_ | PH | P4 | 285.59 | 271.12 |
| F_1_ | SL | P1 | 0.19 | 0.15 |
| F_1_ | SL | P2 | 0.53 | 0.32 |
| F_1_ | SL | P3 | 0.75 | 0.57 |
| F_1_ | SL | P4 | 0.22 | 0.01 |
| F_1_ | SW | P1 | 7.31 | 9.49 |
| F_1_ | SW | P2 | 24.25 | 8.70 |
| F_1_ | SW | P3 | 103.01 | 40.09 |
| F_1_ | SW | P4 | 30.76 | -28.18 |
| F_1_ | NS | P1 | 1.43 | 2.24 |
| F_1_ | NS | P2 | 6.23 | 2.17 |
| F_1_ | NS | P3 | 31.81 | 16.67 |
| F_1_ | NS | P4 | 7.28 | -8.36 |
| F_1_ | NGS | P1 | 23.74 | -1.52 |
| F_1_ | NGS | P2 | 7.89 | 1.59 |
| F_1_ | NGS | P3 | 22.44 | 1.65 |
| F_1_ | NGS | P4 | 32.94 | 4.32 |
| F_1_ | TKW | P1 | 18.52 | 15.44 |
| F_1_ | TKW | P2 | 2.65 | -0.74 |
| F_1_ | TKW | P3 | 13.37 | 9.67 |
| F_1_ | TKW | P4 | 11.63 | -0.20 |
| F_1_ | GY | P1 | 2.26 | 6.47 |
| F_1_ | GY | P2 | 6.97 | 4.13 |
| F_1_ | GY | P3 | 45.62 | 28.57 |
| F_1_ | GY | P4 | 6.35 | -11.99 |
| F_1_ | BIO | P1 | 75.22 | 55.01 |
| F_1_ | BIO | P2 | 46.52 | 6.46 |
| F_1_ | BIO | P3 | 286.20 | 126.11 |
| F_1_ | BIO | P4 | 87.97 | -52.17 |
| F_2_ | PH | P1 | 19.22 | 39.48 |
| F_2_ | PH | P2 | 45.73 | 40.50 |
| F_2_ | PH | P3 | 31.81 | 52.00 |
| F_2_ | PH | P4 | 65.04 | 63.78 |
| F_2_ | SL | P1 | 0.26 | 0.43 |
| F_2_ | SL | P2 | 0.16 | 0.34 |
| F_2_ | SL | P3 | 0.19 | 0.31 |
| F_2_ | SL | P4 | 0.23 | 0.37 |
| F_2_ | SW | P1 | 0.44 | -0.05 |
| F_2_ | SW | P2 | 0.67 | -0.03 |
| F_2_ | SW | P3 | 0.77 | -0.39 |
| F_2_ | SW | P4 | 1.13 | 0.57 |
| F_2_ | NS | P1 | 0.06 | 0.10 |
| F_2_ | NS | P2 | 0.09 | 0.08 |
| F_2_ | NS | P3 | 0.26 | 0.04 |
| F_2_ | NS | P4 | 0.45 | 0.33 |
| F_2_ | NGS | P1 | 2.95 | 5.65 |
| F_2_ | NGS | P2 | 20.20 | 21.26 |
| F_2_ | NGS | P3 | 21.43 | 21.08 |
| F_2_ | NGS | P4 | 12.71 | 19.76 |
| F_2_ | TKW | P1 | 26.06 | -47.72 |
| F_2_ | TKW | P2 | 10.37 | -0.62 |
| F_2_ | TKW | P3 | 92.90 | 94.86 |
| F_2_ | TKW | P4 | 105.65 | 16.52 |
| F_2_ | GY | P1 | 1.15 | -0.47 |
| F_2_ | GY | P2 | 1.58 | -0.67 |
| F_2_ | GY | P3 | 0.03 | 0.10 |
| F_2_ | GY | P4 | 2.42 | 0.91 |
| F_2_ | BIO | P1 | 3.20 | 0.23 |
| F_2_ | BIO | P2 | 0.46 | 0.36 |
| F_2_ | BIO | P3 | 4.02 | 1.48 |
| F_2_ | BIO | P4 | 0.66 | 0.45 |
